# Supplementary material for: Exceptional thermoelectric properties of flexible organic−inorganic hybrids with monodispersed and periodic nanophase
Source: Nat Commun. 2018 Sep 19;9:3817. doi: 10.1038/s41467-018-06251-9 (PMC6145921; doi:10.1038/s41467-018-06251-9)
Supplement: Supplementary file 1 — Supplementary Information [file 41467_2018_6251_MOESM1_ESM.docx]

**Exceptional thermoelectric properties of flexible organic-inorganic hybrids with monodispersed and periodic nanophase**

Wang et al.

**Supplementary Information**

**Exceptional thermoelectric properties of flexible organic-inorganic hybrids with monodispersed and periodic nanophase**

Liming Wang^1^, Zimeng Zhang^1^, Yuchen Liu^1^, Biran Wang^1^, Lei Fang^2^, Jingjing Qiu^3^, Kun Zhang^4^ and Shiren Wang^1🟉^

^1^Department of Industrial and Systems Engineering, Texas A&M University, College Station, Texas 77843, USA

^2^Department of Chemistry, Texas A&M University, College Station, Texas 77843, USA

^3^Department of Mechanical Engineering, Texas Tech University, Lubbock, TX 77409, USA

^4^College of Textiles, Donghua Univeristy, Shanghai, 201620, China

^🟉^e-mail: [s.wang@tamu.edu](mailto:s.wang@tamu.edu)


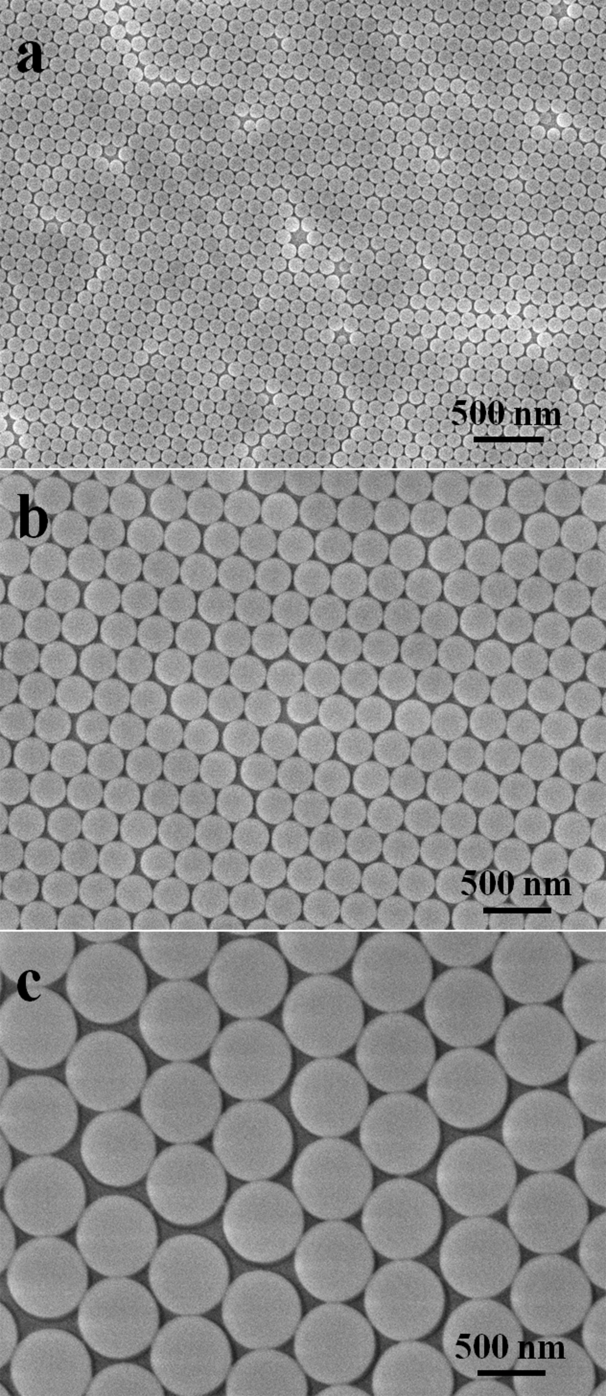


**Supplementary Fig. 1 |** Morphology of polystyrene (PS) nanospheres. SEM images of closely packed monolayer PS nanospheres with diameter of (a) 100 nm, (b) 300 nm, and (c) 600 nm.


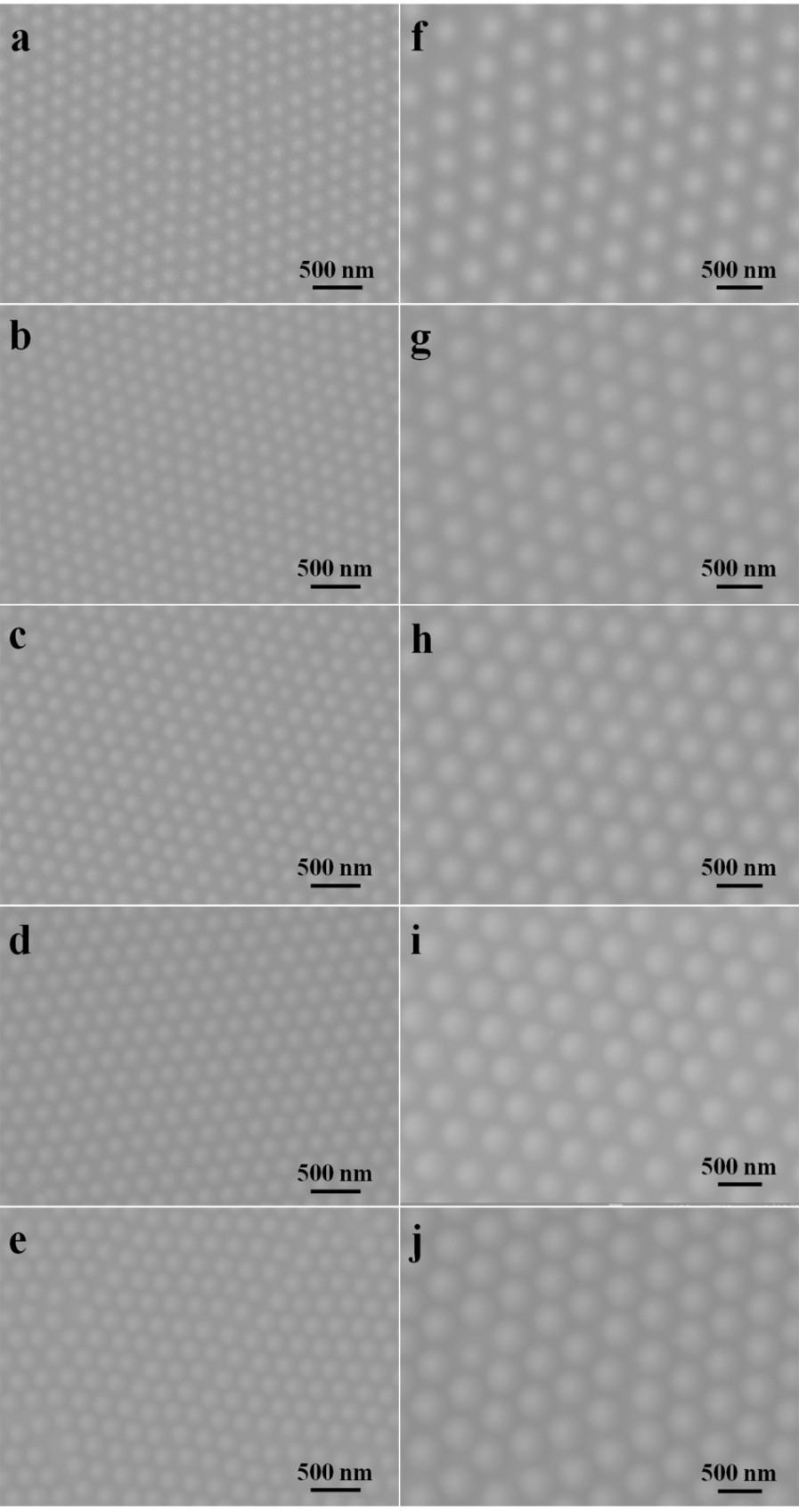


**Supplementary Fig. 2 |** Morphology of hybrid films. SEM images of prepared (a-e) PEDOT/Bi_2_Te_3_(300) hybrid films and (f-j) PEDOT/Bi_2_Te_3_(600) hybrid films.


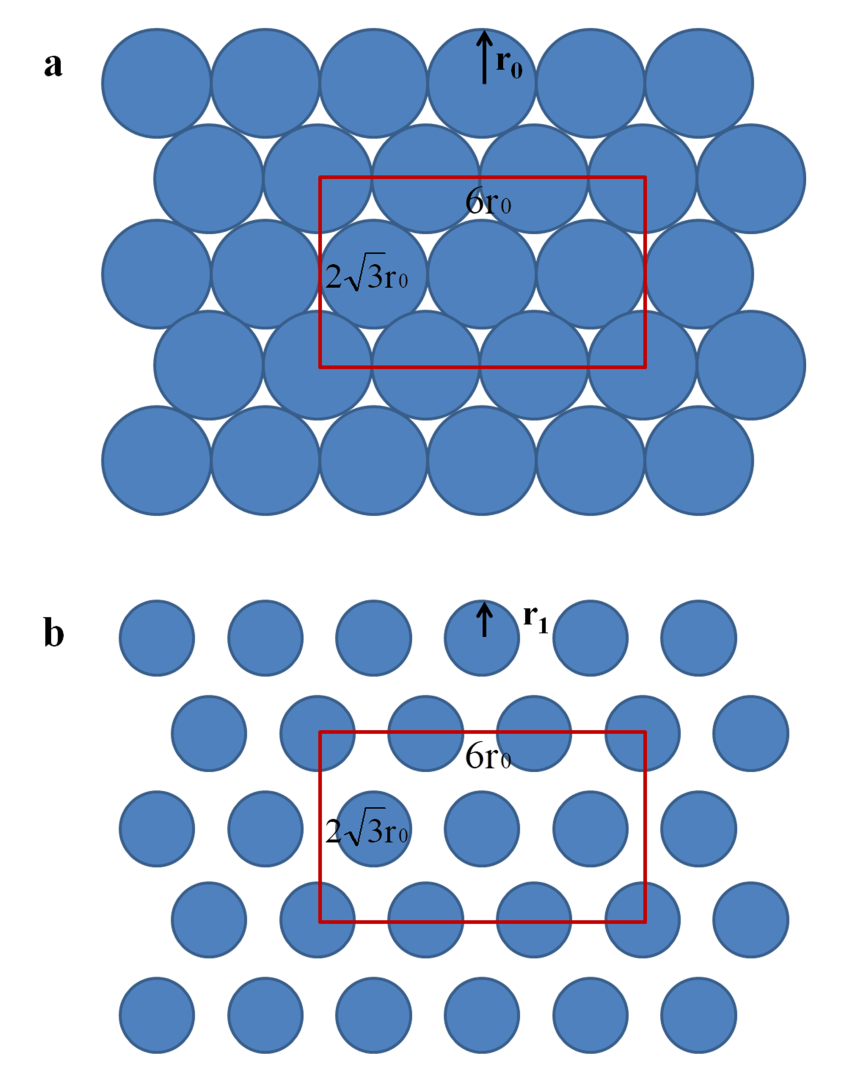


**Supplementary Fig. 3 |** Schematic diagram. (a) Closely packed PS nanospheres and (b) obtained Bi_2_Te_3_ nanoparticle array.


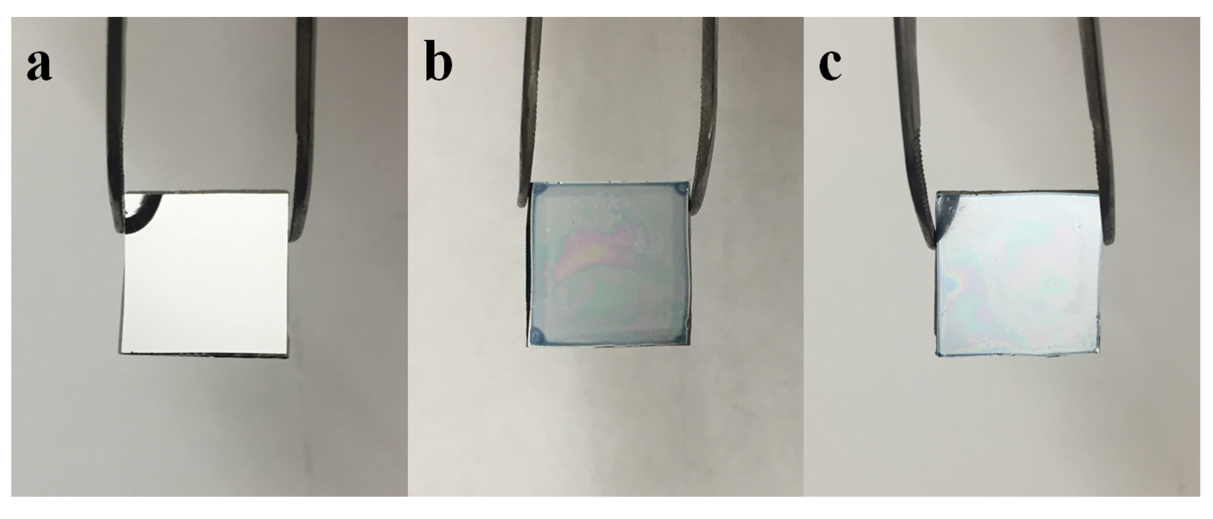


**Supplementary Fig. 4 |** Digital photos. (a) Bi_2_Te_3_ film, (b) PEDOT film, and (c) PEDOT/Bi_2_Te_3_ hybrid film on Si substrates.


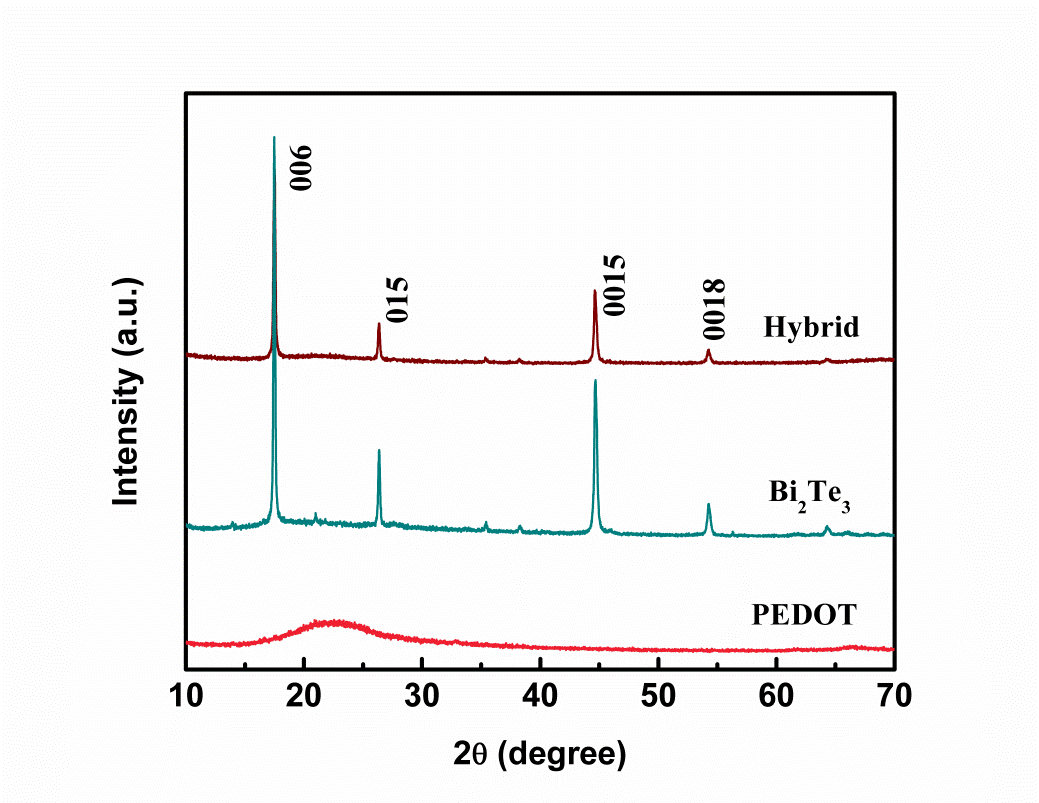


**Supplementary Fig. 5 |** XRD patterns. The XRD pattern of PEDOT film exhibits a broad diffraction peak due to the amorphous structure. The detected peaks of both Bi_2_Te_3_ film and PEDOT/Bi_2_Te_3_(100) hybrid film with ~31 vol% Bi_2_Te_3_ nanoparticle fraction can be well indexed to the standard card of Bi_2_Te_3_ (JCPDS card no. 15-0863)^1^.


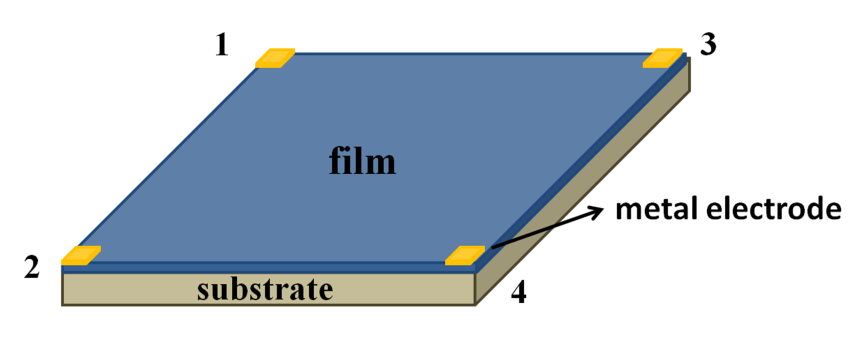


**Supplementary Fig. 6 |** Illustration of the sample configuration for electrical conductivity measurement.


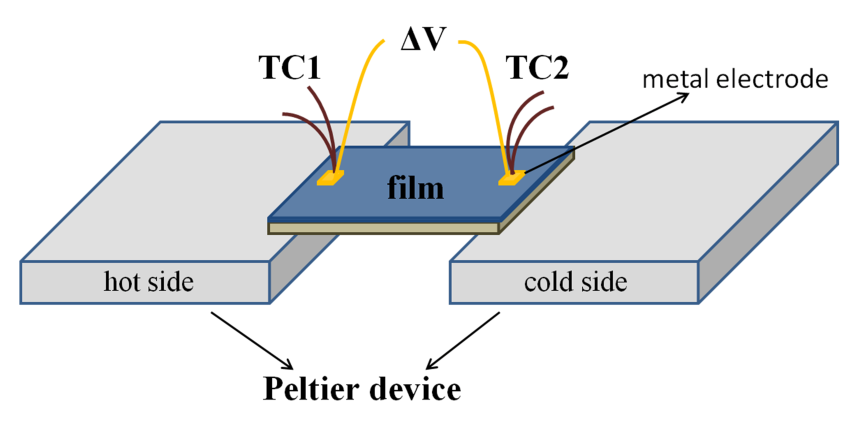


**Supplementary Fig. 7 |** Schematic of the Seebeck coefficient measurement apparatus.


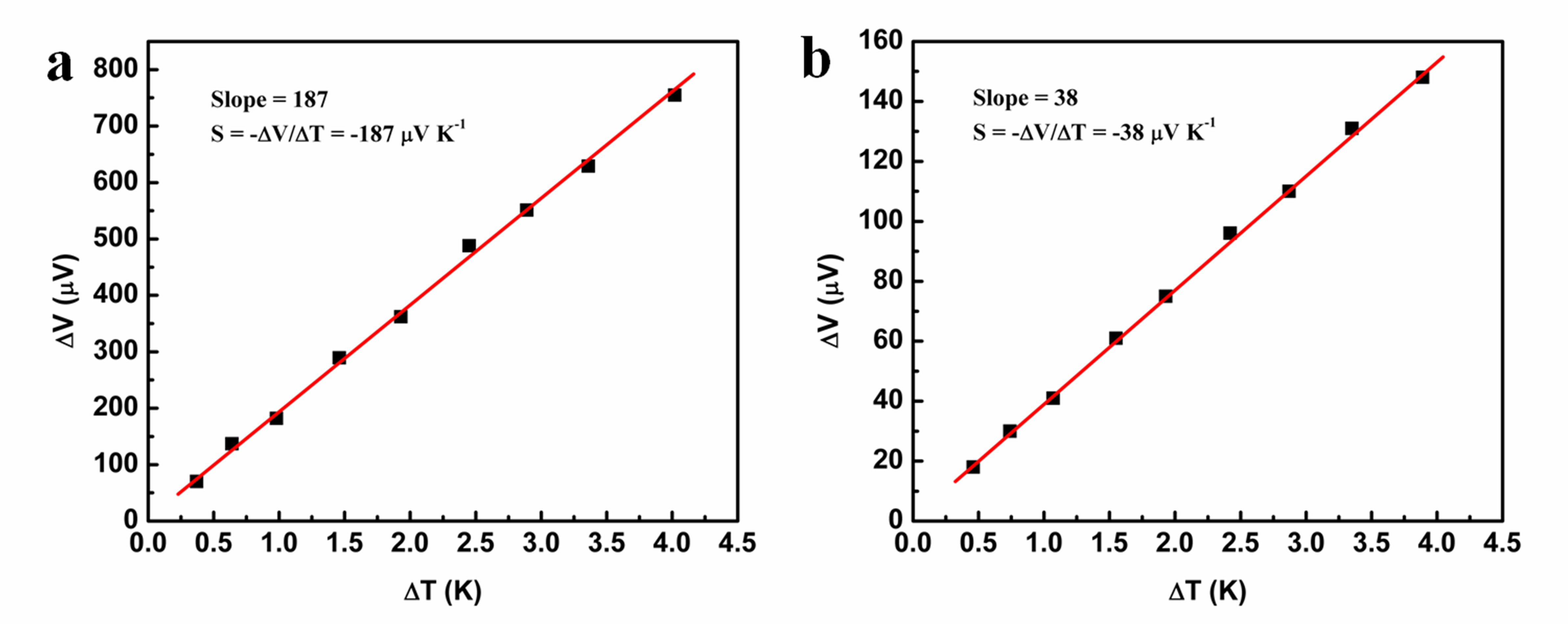


**Supplementary Fig. 8 |** The measured Seebeck coefficient of standard samples. (a) N-type Bi_2_Te_3_ and (b) constantan.


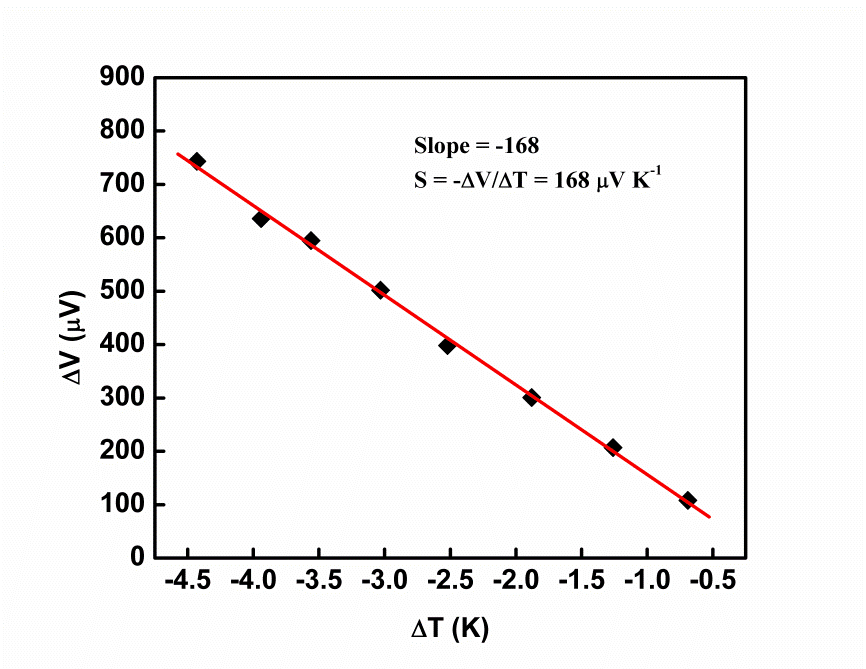


**Supplementary Fig. 9 |** A typical Seebeck coefficient measurement curve. The sample isPEDOT/Bi_2_Te_3_(100) hybrid film with ~31 vol% Bi_2_Te_3_.


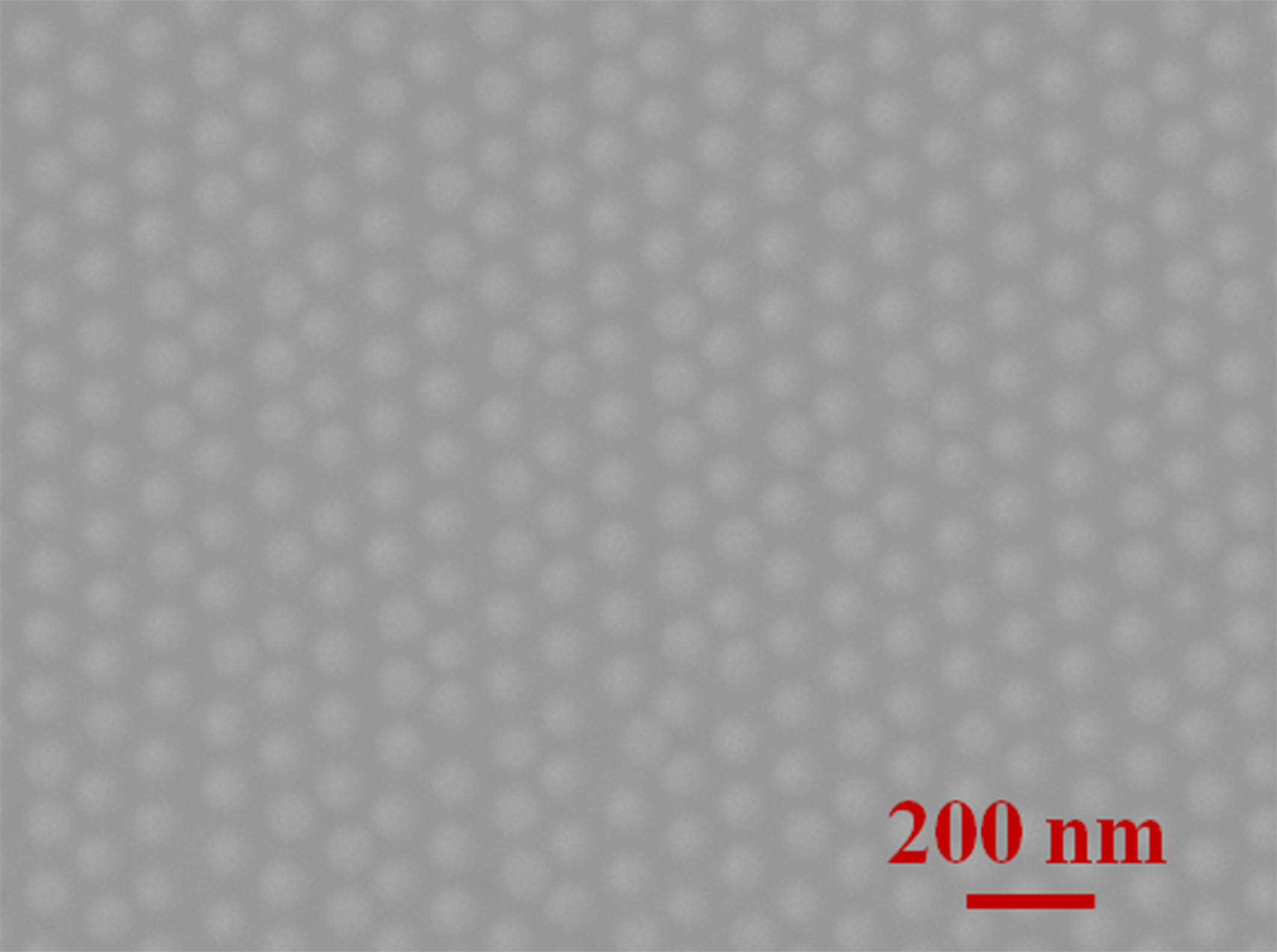


**Supplementary Fig.10 |** SEM image of prepared PEDOT/31 vol% Bi_2_Te_3_(100) hybrid thick film.


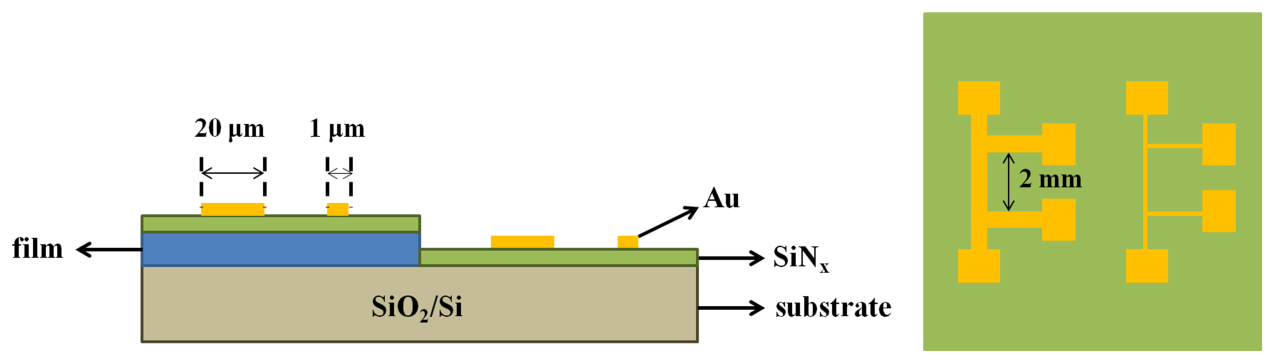


**Supplementary Fig. 11 |** Schematic illustration. The device structure and electrodes used for the thermal conductivity measurement by differential 3ω method.


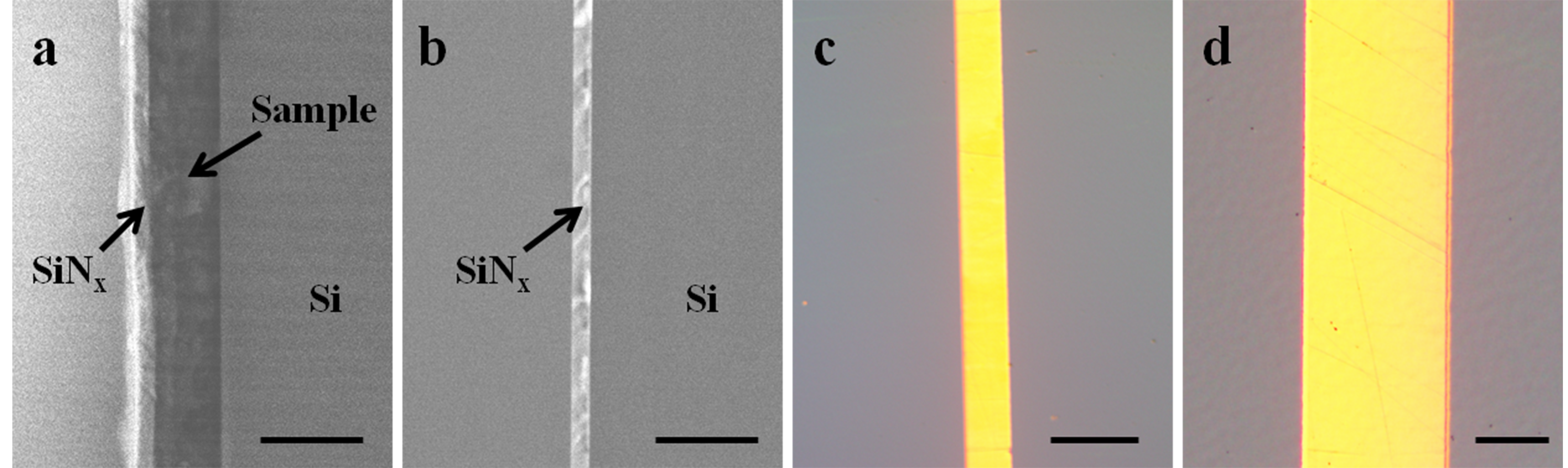


**Supplementary Fig. 12 |** Morphology of the device for the thermal conductivity measurement. (a, b) Cross-sectional SEM images of device for the thermal conductivity measurement, (a) sample and (b) reference. (c, d) Optical images of gold line with width of (c) 1 μm and (d) 20 μm. The scale bars in a, b, c and d are 1 μm, 1 μm, 2 μm and 10 μm, respectively.


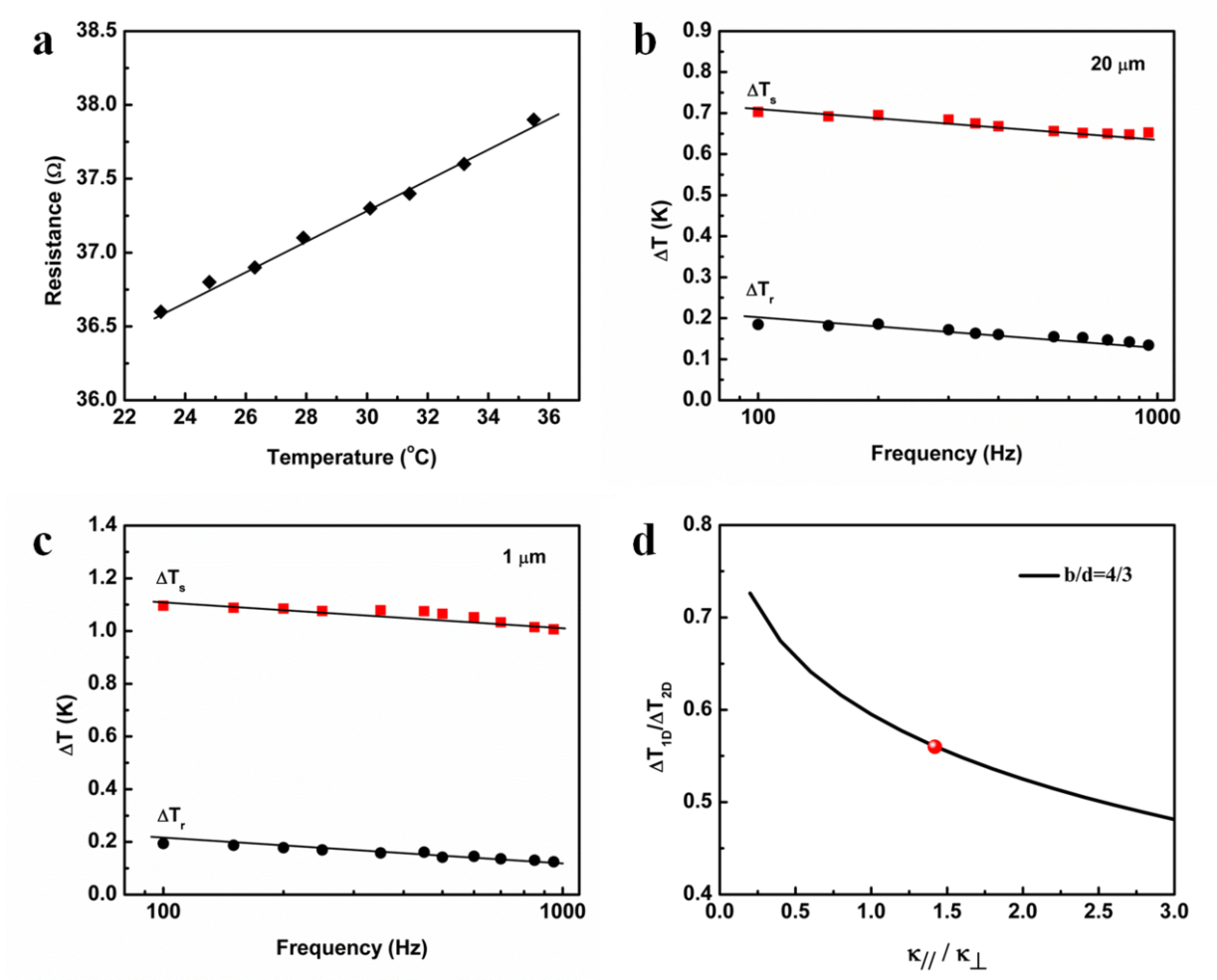


**Supplementary Fig. 13 |** Typical results of thermal conductivity measurement. (a) Temperature coefficient of resistance of the metal line heater. (b) Temperature oscillation amplitudes of 20 μm line heater for measurements of Δ*T_1D_*. (c) Temperature oscillation amplitudes of 1 μm line heater for measurements of Δ*T_2D_*. (d) The relationship between Δ*T_1D_*/Δ*T_2D_* and *κ*_//_/*κ*_⊥_ under *b*/*d* = 4/3.

**Supplementary Fig. 14 |** Comparisons of power factor and *ZT* of prepared PEDOT film, PEDOT/Bi_2_Te_3_ hybrid films with ~31 vol% Bi_2_Te_3_, and Bi_2_Te_3_ film.





**Supplementary Fig. 15 |** Air stability test. The prepared PEDOT/Bi_2_Te_3_(100) hybrid film (square), PEDOT/Bi_2_Te_3_(300) hybrid film (pentacle), and PEDOT/Bi_2_Te_3_(600) hybrid film (circle) with ~31 vol% Bi_2_Te_3_ were relatively stable in air at room temperature, displaying slightly changes in electrical conductivity after one week.


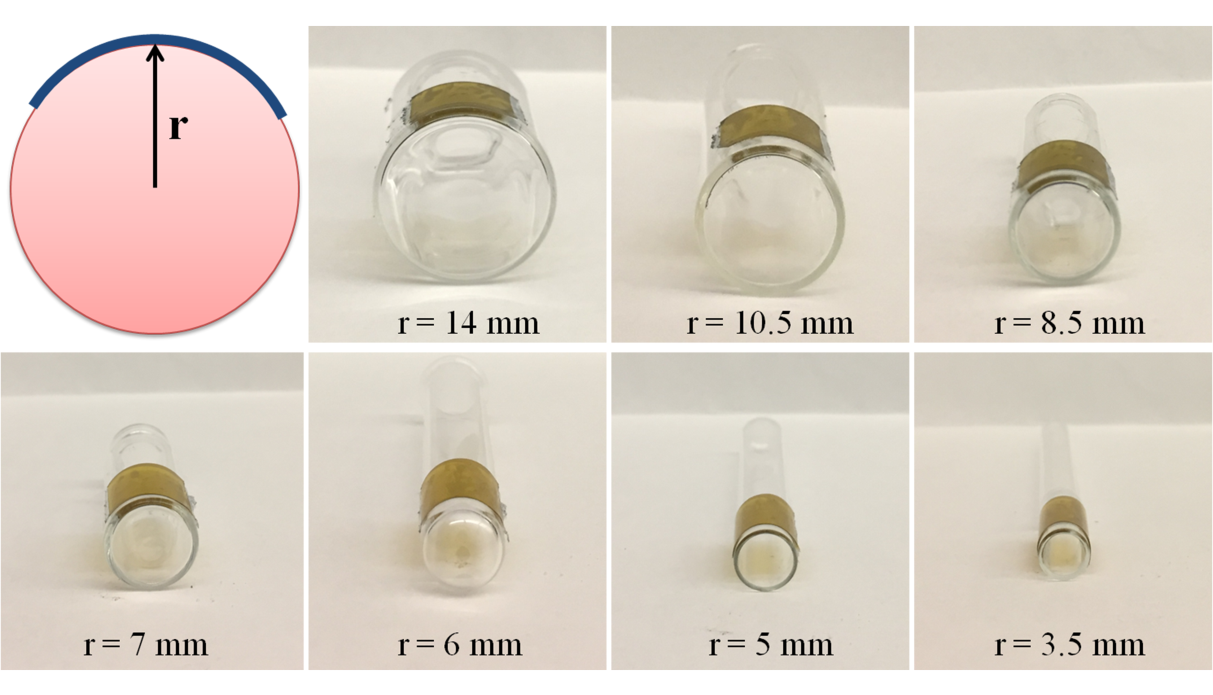


**Supplementary Fig. 16 |** Digital photos. Flexible PEDOT/Bi_2_Te_3_(100) hybrid films with ~31 vol% Bi_2_Te_3_ under different curve radius *r*.


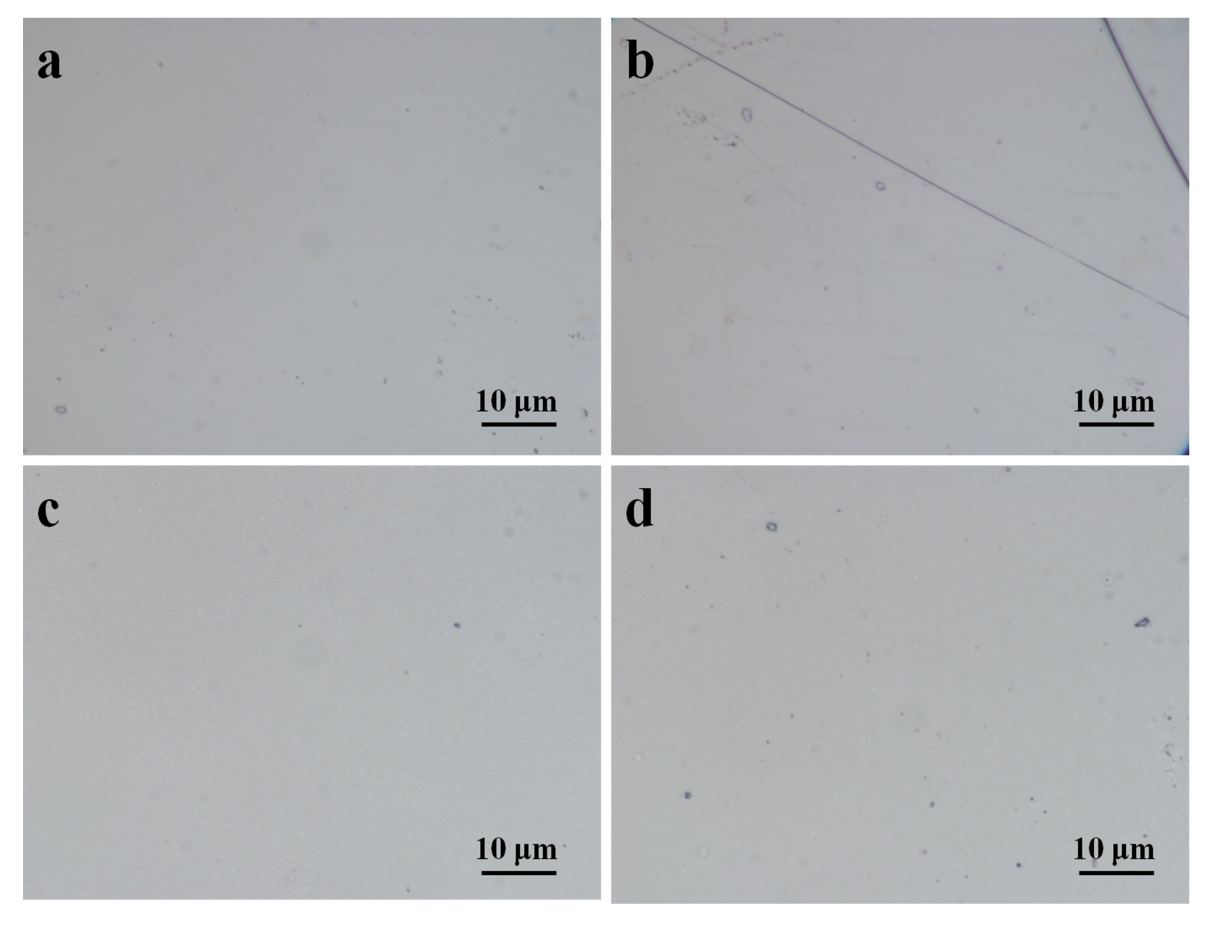


**Supplementary Fig. 17 |** Optical images. (a,b) Flexible Bi_2_Te_3_ film and (c,d) PEDOT /Bi_2_Te_3_(100) hybrid films with ~31 vol% Bi_2_Te_3_. (a,c) Before bending. (b,d) After 100 times of bending at a curve radius of 3.5 mm.


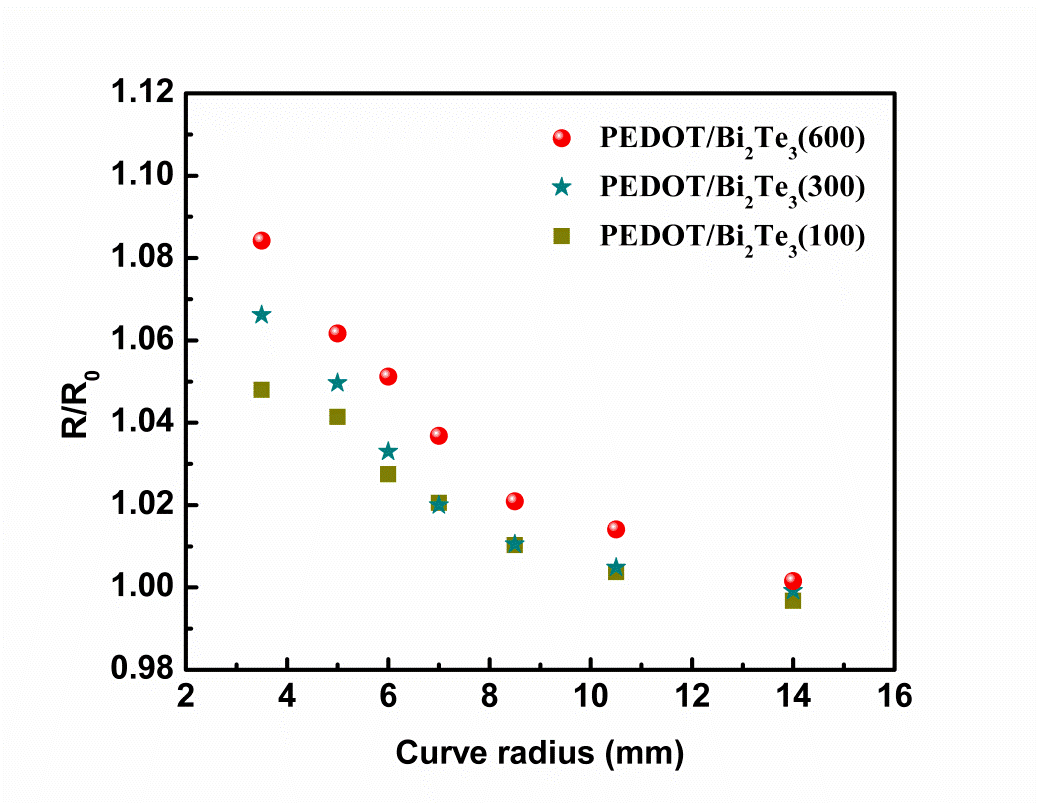


**Supplementary Fig. 18 |** Stability of electrical conductivity. Changes of resistance for PEDOT/Bi_2_Te_3_ hybrid films with ~31 vol% Bi_2_Te_3_ nanoparticle fraction as a function of curve radius *r*.


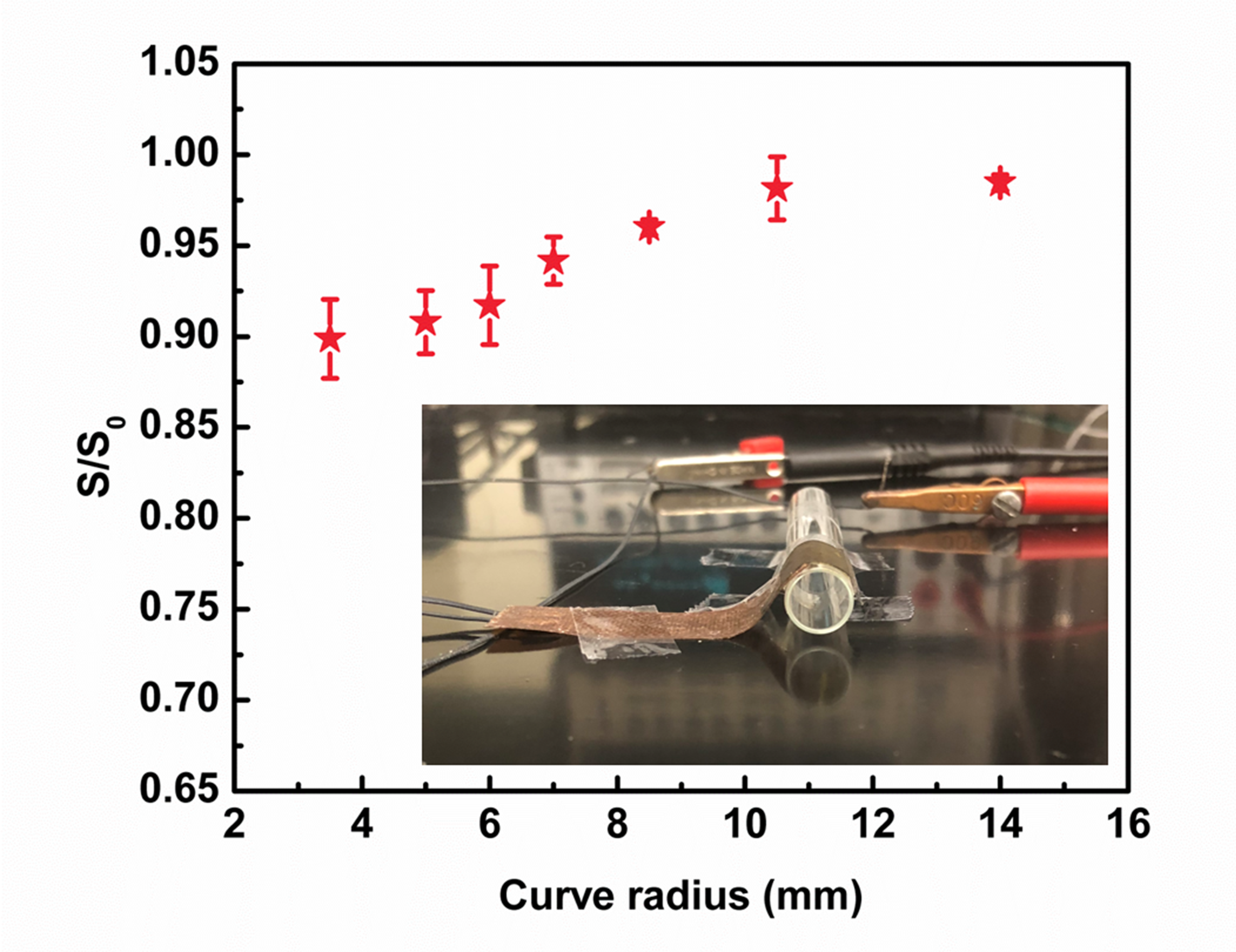


**Supplementary Fig. 19 |** Stability of Seebeck coefficient. The Seebeck coefficient *S* of prepared PEDOT/Bi_2_Te_3_(100) hybrid film with ~31 vol% Bi_2_Te_3_ nanoparticle fraction as a function of curve radius, where *S_0_* is the Seebeck coefficient before bending. Each points show the standard deviations from three independent measurements. Temperature difference was produced by heating one side of the hybrid film with a flexible heater which was connected with a Keithley 2400 SourceMeter. The Seebeck coefficient kept stable upon different curve radius, only ~10% changes even at a very low curve radius of 3.5 mm.

**Supplementary Table 1 |** Room-temperature thermoelectric properties of Bi_2_Te_3_ film on SiO_2_/Si substrate prepared under the same condition for hybrid films.

| Electrical conductivity  (S cm^-1^) | Seebeck  coefficient  (μV K^-1^) | Thermal  conductivity  (W m^-1^ K^-1^) | *ZT* |
| --- | --- | --- | --- |
| 143 | 273 | 0.82 | 0.39 |

**Supplementary Table 2 |** The detailed room-temperature thermoelectric properties for the state-of-the-art organic materials and organic/inorganic hybrids listed in Fig. 4d.

| Materials | Electrical  conductivity  (S cm^-1^) | Seebeck  coefficient  (μV K^-1^) | Thermal  conductivity  (W m^-1^ K^-2^) | *ZT* | Reference in the main text |
| --- | --- | --- | --- | --- | --- |
| PEDOT-Tos | 80 | 200 | 0.37 | 0.25 | [17] |
| Poly[K_x_(Ni-ett)] | 46 | -122 | 0.21 | 0.1 | [37] |
| PEDOT:PSS | 880 | 73 | 0.336 | 0.42 | [34] |
| TiS_2_/organic | 790 | -78 | 0.69 | 0.2 | [5] |
| Poly(Ni-ett) | 230 | -125 | 0.5 | 0.21 | [38] |
| PEDOT:PSS/SnSe | 320 | 110 | 0.36 | 0.32 | [9] |
| Polymer/SWNTs | 170 | 64 | 1.7 | 0.012 | [35] |
| C_6_H_4_NH_2_CuBr_2_I | 3600 | -70 | 3.63 | 0.14 | [39] |
| PVDF/Ni | 4701 | -21 | 0.55 | 0.11 | [6] |
| PEI doped SWNTs | 1780 | -82 | 18 | 0.02 | [36] |
| **PEDOT/Bi_2_Te_3_** | **483** | **168** | **0.71** | **0.58** | **This work** |

**Supplementary Note 1**

**Estimation of the Bi_2_Te_3_ nanoparticle fraction in hybrid films**

As shown in Supplementary Fig. 3, the center of the PS nanospheres would not change during RIE treatment. Based on the periodic structure of Bi_2_Te_3_ nanoparticles, the volume fraction (*V_d_*) of the Bi_2_Te_3_ nanoparticles in hybrid film can be estimated by,

$V_{d}=\frac{\pi*r_{1}^{2}*d_{1}}{2\sqrt{3}*r_{0}^{2}*d_{0}}$, (1)

where *r_0_* is the initial radius of PS nanosphere, *r_1_* is the radius of obtained Bi_2_Te_3_ nanoparticle, *d_0_* is the thickness of hybrid films, and *d_1_* is the thickness of Bi_2_Te_3_ nanoparticle. The thickness of Bi_2_Te_3_ nanoparticle (*d_1_*) can be accurately controlled by the monitor of thermal evaporation instrument, which is also confirmed by a Bruker DektakXT surface profiler. The thickness of Bi_2_Te_3_ nanoparticle (*d_1_*) is ~70 nm. The thickness of hybrid films (*d_0_*) is mainly determined by the spin coating conditions of the VPP process. For hybrid films, we measured the film thickness in at least five different areas for each sample with a Bruker DektakXT surface profiler. The thickness of hybrid films (*d_0_*) are ~75 nm. The Bi_2_Te_3_ nanoparticle fraction in hybrid films is estimated with the tested film thickness.**Supplementary Note 2**

**Electrical conductivity measurement**

The electrical conductivity was measured based on the Van der Pauw method with a home-made apparatus^2,3^. As shown in Supplementary Fig. 6, electrodes were made by pressing indium on four corners of the thin film. Four probes were pressed on the electrodes. *I-V* sweep was performed using a Keithley 6221 as constant current source and a Keithley 2182A as voltage meter. The apparatus was calibrated with a standard resistance of 200 Ω before the measurement of thin films. The film thickess was tested with a Bruker DektakXT surface profiler.

**Supplementary Note 3**

**Seebeck coefficient measurement**

Supplementary Fig. 7 shows the schematic of the Seebeck coefficient measurement apparatus. Peltier devices were used to create temperature difference between the two sides of thin film by applying an electrical current. A set of parallel and narrow line-shaped (1 mm × 7 mm) gold electrodes with thickness of 150 nm and spacing of 10 mm were thermally deposited onto the sample, in order to get an accurate determination of the actual thermoelectric voltage (Δ*V*)^2,4^. Two T-type thermocouples (TC1, TC2) with diameter of 127 µm were placed on the sample to determine the temperature difference (Δ*T*). Both the thermocouples and the sample surface in the region of the thermocouples were erased by a swab with ethanol each time in order to avoid the contact problem and get accurate measurement in temperature difference. The Seebeck coefficient was obtained from the slope of the linear Δ*V-*Δ*T* curve. In order to confirm the accuracy of this method, two different reference samples, n-type Bi_2_Te_3_ sample with Seebeck coefficient of -180 µV K^-1^ and constantan with Seebeck coefficient of -36 µV K^-1^ which were obtained by a well-calibrated commercial instrument ZEM-3, were tested. The Seebeck coefficient measurement curves of this two samples are illustrated in Supplementary Fig. 8. It reveals the measurement errors for Seebeck coefficient were less than 6%.

**Supplementary Note 4**

**Thermal conductivity measurement**

As mentioned above, the thickness of as-prepared hybrid films are around 75 nm. Although the electrical conductivity and Seebeck coefficient were measured from these thin films, it is still very difficult to perform the in-plane thermal conductivity measurement of these thin films with thickness lower than 100 nm^5,6^. Thus we prepared thick hybrid films (thickness of ~0.75 µm) for thermal conductivity measurement by a 3*ω* method^5-7^, in order to estimate the *ZT* values. Compared with the method for preparing thin hybrid films, several modifications were made to prepare thick hybrid films. Firstly, 1 µm thick SiO_2_ layer was deposited on the Si wafer via PEVCD in the step of “pre-treatment of sustrates”. Then, the etching time for removing the exposed SiO_2_ by RIE was increased to 30 min in the step of “fabrication of Bi_2_Te_3_ nanoparticle arrays”. Also, the thickness of deposited Bi_2_Te_3_ film was fixed to 0.7 µm. Finally, the parameters of spin coating in the step of VPP was changed to 1500 rpm for 25 s. The VPP process was repeated for three times to obtain desired thick hybird films. The total thermal conductivity contains the lattice thermal conductivity and electronic thermal conductivity. In our hybrid films, the lattice thermal conductivity is mainly dominated by the film morphology. The thick films for the 3*ω* thermal conductivity measurement display a very similar morphology as compared to the corresponding thin films. For example, the SEM image of as-prepared PEDOT/31 vol% Bi_2_Te_3_(100) hybrid thick film is given in Supplementary Fig. 10. While the electronic thermal conductivity is related to the electrical conductivity. Our thick films for thermal conductivity measurement only possesses 10~20% lower electrical conductivity as compared to the corresponding thin films. For example, the electrical conductivity of PEDOT/31 vol% Bi_2_Te_3_(100) hybrid thick film is ~391 S cm^-1^, which is ~483 S cm^-1^ for the corresponding thin film. It may not result in a relatively large error for the electronic thermal conductivity. Therefore, the total thermal conductivity of thick film may have limited deviation from the thin one. As to the fabrication of PEDOT thick films by VPP method for thermal conductivity measurement, the spin coating was also conducted at 1500 rpm for 25 s. The VPP process was repeated for three times to obtain desired PEDOT thick films. The thickness of as-prepared PEDOT thick film was measured to be ~0.62 µm. The schematic diagrams of the device structure and electrodes used for 3*ω* measurement are shown in Supplementary Fig. 11. Firstly, 100 nm thick SiN_x_ insulating layer was deposited on the film by PECVD. Then, gold line heaters with length of 2 mm, width of 1 μm/20 μm, and thickness of 100 nm were patterned by using photolithography method (SUSS MA-6 Mask Aligner). Cr layer with thickness of 5 nm was deposited between film and gold line heater in order to improve the adhesion.

When the width of the gold line is 20 μm which is much larger than the thickness of film (~0.75 μm), the thermal conduction is nearly one dimensional in the direction vertical to the film. In this situation, we can obtain the cross-plane thermal conductivity of film. The temperature drop in the film (Δ*T_f_*) can be given by,

$\frac{\Delta T_{f}}{P_{s}}=\frac{\Delta T_{s}}{P_{s}}-\frac{\Delta T_{r}}{P_{r}}$, (2)

where Δ*T_s_* and Δ*T_r_* are the temperature oscillations of the gold line heaters on the sample including all layers and the reference, *P_s_* and *P_r_* are the corresponding power dissipations. Δ*T_s_* and Δ*T_r_* are determined by,

$\Delta\text{T}_{s,r}=2R\frac{dT}{dR}\frac{V_{3\omega}}{V_{1\omega}}$, (3)

where *R* and *dT/dR* are the resistance and temperature coefficient of resistance of the gold line heater, *V_3ω_* and *V_1ω_* are the first and third harmonic voltages. The cross-plane thermal conductivity of the film is calculated by,

$\kappa_{\perp}=\frac{Pd_{f}}{bL\Delta T_{f}}$, (4)

where *d_f_* is the film thickness, *b* is the width of the heater line, and *L* is the length of the heater line.

When the width of the gold line is 1 μm which is close to the thickness of film, the heat transport is sensitive to both cross-plane and in-plane thermal conductivity of the film. The ratio of reduced temperatures related to the narrow (Δ*T_2D_*) and the wide (Δ*T_1D_*) gold heater lines can be shown as,

$\frac{\Delta T_{2D}}{\Delta T_{1D}}=\left(\frac{\kappa_{\perp}}{\kappa_{//}}\right)^{1/2}\frac{b_{n}}{d_{f}}\frac{K\left(\lambda\right)}{2K'\left(\lambda\right)}$, (5)

where *b_n_* is the width of the narrow gold heater line, *K(λ)* is the complete elliptical integral of the first kind, and *K'(λ)* is its complementary integral. The argument *λ* is given by,

$\frac{1}{\lambda}=\text{cosh}\left[\frac{\pi}{4}\frac{b_{n}}{d_{f}}\left(\frac{\kappa_{\perp}}{\kappa_{//}}\right)^{1/2}\right]$. (6)

Supplementary Fig. 13 gives an example for testing the in-plane thermal conductivity of PEDOT/Bi_2_Te_3_ hybrid film with ~31 vol% Bi_2_Te_3_. For the wide metal line heater (20 μm), Δ*T_f_* was calculated as ~0.51 K. The vertical thermal conductivity was 0.54 W m^-1^ K^-2^ based on equation S4. For the narrow metal line heater (1 μm), Δ*T_f_* was calculated as ~0.91 K. The ratio of Δ*T_1D_*/Δ*T_2D_* was 0.56 and fitted to equation S5, determining the value of *κ*_//_/*κ*_⊥_ to be 1.42. Thus, *κ*_//_ was 0.77 W m^-1^ K^-2^.

**Supplementary References**

1. He, X. et al. PVP-Assisted Solvothermal Synthesis of High-Yielded Bi2Te3 Hexagonal Nanoplates: Application in Passively Q-Switched Fiber Laser. *Sci. Rep.* **5**, 15868 (2015).

2. Zhang, K., Qiu, J. J. & Wang, S. R. Thermoelectric properties of PEDOT nanowire/PEDOT hybrids. *Nanoscale* **8**, 8033-8041 (2016).

3. Zhang, K. et al. Effect of host-mobility dependent carrier scattering on thermoelectric power factors of polymer composites. *Nano Energy* **19**, 128-137 (2016).

4. Reenen, S. V. & Kemerink, M. Correcting for contact geometry in Seebeck coefficient measurements of thin film devices. *Org. Electron.* **15**, 2250-2255 (2014).

5. Kim, G. H., Shao, L., Zhang, K. & Pipe, K. P. Engineered doping of organic semiconductors for enhanced thermoelectric efficiency. *Nat. Mater.* **12**, 719-723 (2013).

6. Huang, D. et al. Conjugated-Backbone Effect of Organic Small Molecules for n-Type Thermoelectric Materials with ZT over 0.2. *J. Am. Chem. Soc.* **139**, 13013-13023 (2017).

7. Bubnova, O. et al. Optimization of the thermoelectric figure of merit in the conducting polymer poly(3,4-ethylenedioxythiophene). *Nat. Mater.* **10**, 429-433 (2011).
